# Tailoring the Anodic Hafnium Oxide Morphology Using Different Organic Solvent Electrolytes

**DOI:** 10.3390/nano10020382

**Published:** 2020-02-22

**Authors:** Arlete Apolinário, Célia T. Sousa, Gonçalo N. P. Oliveira, Armandina M. L. Lopes, João Ventura, Luísa Andrade, Adélio Mendes, João P. Araújo

**Affiliations:** 1 Instituto de Física de Materiais Avançados, Nanotecnologia e Fotónica (IFIMUP), Departamento de Física e Astronomia, Faculdade de Ciências, Universidade do Porto, Rua do Campo Alegre, 678, 4169-007 Porto, Portugal; celiasousa@fc.up.pt (C.T.S.); goliveira@fc.up.pt (G.N.P.O.); armandina.lopes@fc.up.pt (A.M.L.L.); joventur@fc.up.pt (J.V.); 2 Laboratory for Process Engineering, Environment, Biotechnology and Energy (LEPABE), Departamento de Engenharia Química, Faculdade de Engenharia, Universidade do Porto, R. Dr. Roberto Frias, 4200-465 Porto, Portugal; luisa.andrade@fe.up.pt (L.A.); mendes@fe.up.pt (A.M.)

**Keywords:** anodic hafnium oxide, HfO_2_, anodic oxide, anodization, nanotubes, nanoporous, organic solvent, dielectric, viscosity

## Abstract

Highly ordered anodic hafnium oxide (AHO) nanoporous or nanotubes were synthesized by electrochemical anodization of Hf foils. The growth of self-ordered AHO was investigated by optimizing a key electrochemical anodization parameter, the solvent-based electrolyte using: Ethylene glycol, dimethyl sulfoxide, formamide and N-methylformamide organic solvents. The electrolyte solvent is here shown to highly affect the morphological properties of the AHO, namely the self-ordering, growth rate and length. As a result, AHO nanoporous and nanotubes arrays were obtained, as well as other different shapes and morphologies, such as nanoneedles, nanoflakes and nanowires-agglomerations. The intrinsic chemical-physical properties of the electrolyte solvents (solvent type, dielectric constant and viscosity) are at the base of the properties that mainly affect the AHO morphology shape, growth rate, final thickness and porosity, for the same anodization voltage and time. We found that the interplay between the dielectric and viscosity constants of the solvent electrolyte is able to tailor the anodic oxide growth from continuous-to-nanoporous-to-nanotubes.

## 1. Introduction

Advances in nanoscience and nanotechnology are interconnected with the development of new platforms where the physical properties of materials/structures, like size, porosity, geometry and surface functionalization can be controlled at the nanoscale. In this way, the potential of applications is created for a large number of areas [1,2,3,4], and thus, are pushing fast the research on the topic. As an example, metal-oxide nanostructures, such as nanotube arrays, have been instigating great interest, due to their demand for optoelectronics, microelectronics, energy storage, solar cells, catalysis or biomedical applications [1,2,3,4,5,6]. 

Hafnium oxide (HfO_2_) with its high thermal, chemical and mechanical stability, as well as its high refractive index and dielectric constant is remarkably appealing for new nanostructure architectures like nanoporous or nanotube (NT) arrays and a large range of applications [5,6,7,8,9,10,11,12]. Having into account the emerging application of anodic TiO_2_ nanotubes in DSCs, the question arises about the applicability of self-ordered arrays of anodic HfO_2_ for the same purpose. The truth is that the use of an HfO_2_ compact layer on dye-sensitized solar cells (DSCs) results in improved photovoltaic performance of 66%, compared to DSCs with a conventional sol-gel processed TiO_2_ layer [8].

Self-organized porous anodic hafnium oxide (AHO) layers were first successfully obtained by Schmuki et al. via the electrochemical anodization of hafnium foils [13]. Using 50 V in a 1 M H_2_SO_4_ +0.2 wt% NaF electrolyte at room temperature, high-aspect-ratios AHO nanoporous templates with several tens of micrometers in thickness were obtained. The pore diameter increased with the anodization potential, where the latter was a factor that affected the morphology and the structure of the porous oxide. On the other hand, highly ordered HfO_2_ NT arrays were successfully realized through electrochemical anodizations in NH_4_F and ethylene glycol-based electrolytes [14,15]. Such realization largely benefited from the developments obtained in the production of self-ordered TiO_2_ NTs arrays.

Recent developments in electrochemical anodization techniques allow us to prepare a variety of self-organized metal oxide nanotube arrays directly from substrates of value metals, such as hafnium oxide. After the first generation of anodic TiO_2_ NT arrays produced using an aqueous HF based electrolyte, the NT fabrication process has come a long way [16]. The pioneer work of Grimes et al., introducing a variety of organic electrolytes, including ethylene glycol (EG), dimethyl sulfoxide (DMSO), formamide (FA) and N-methylformamide (NMF), was the key to achieve long (hundreds of microns) and ordered TiO_2_ NT arrays [17,18]. The use of organic electrolytes results in a reduced propensity to form an oxide and leads to longer NT arrays. Furthermore, the action of organic-electrolytes lowers the anodic oxide film relative permittivity, and thus, increases its dielectric breakdown voltage and the attainable range of anodization potentials [19]. By mimicking the electrolyte used in TiO_2_ NTs, Qiu et al. obtained self-ordered nanoporous anodic hafnium oxide (AHO) NTs [introducing ethylene glycol (with NH_4_F) based electrolytes] [14,15].

HfO_2_ nanostructures (nanoporous or nanotubes) show promising applications in several fields, such as nanofluidics and electrical engineering systems [20], sensor applications, particularly in real-time bio-sensing [21], as a gate dielectric in place of/or in combination with SiO_2_ in electronic devices, such as field effect transistors [22] or has due to its high melting temperature and excellent physical, electronic and chemical properties or has multifunctional data storage medium [7].

In this work, we investigated the growth of self-ordered AHO nanoporous/nanotubes templates synthesized by the electrochemical anodization of Hf foils. Several organic solvents (EG, DMSO, FA and NMF), combined with fluoride ions, were used to understand the influence of the solvent in the fabrication process of AHO. The electrolyte solvent was found to be a key factor in the morphology and final layer thickness of AHO. Vertically oriented nanoporous and NT arrays were obtained, together with other different shapes and morphologies. We found that the organic solvent used in the electrolyte plays a main role in morphology, and thus, we can engineer different structures, from pores to tubes and also tune the regularity of the self-ordered structures. Additionally, the length of such oxide structures was found to depend on the solvent type, leading to thicknesses of several tens of micrometers. Moreover, a detailed analysis of the growth mechanism and formation stages of such structures was extracted through the anodization (density current vs. anodization time), barrier layer thickness vs. anodization time and charge curves.

## 2. Materials and Methods 

Prior to the anodization, Hf foils (0.127 mm thick, 99.99% purity from AlfaAesar (Heysham, United Kingdom) were cut into 1 cm^2^ pieces, and ultrasonically cleaned—first in ethanol and after in deionized water, 10 min each stage. Afterwards, the foils were electrochemically anodized (as-rolled, without any pre-treatment on the surface) in an in-house made anodization cell (two-electrodes), where Hf acted as the anode and an inert Pt mesh as the cathode [23]. The time evolution of the current density [*j*(t)] was monitored during the anodization process using a Keithley 2004 Sourcemeter (Solon, United States) remotely controlled by a LabView (National Instruments, Austin, United States) application (using a 100 ms acquisition step for the first 5 min). The electrochemical anodization was carried out in four different samples in freshly prepared electrolyte solutions containing NH_4_F (0.3 wt%) (to provide fluoride ions), H_2_O (2 wt%) and different organic solvents: Ethylene glycol (EG), formamide (FA), *N*-methylformamide (NMF) and dimethyl sulfoxide (DMSO). All the anodizations were performed under a constant potential of 60 V for 1 h, at room temperature with mechanical stirring [18,24]. After the anodization, the as-prepared samples were immediately cleaned with ethanol. From now on, the samples prepared with different electrolyte solvent will be referred to as EG, FA, NMF and DMSO. The NTs morphology was evaluated by an FEI Quanta 400FEG Field Emission (Hillsboro, United States) Scanning Electron Microscopy (SEM) using cross-sections (for the AHO length calculation) and surface top views. 

## 3. Results and Discussion

### 3.1. Growth Mechanism: Anodization Curves with Different Organic Solvents

**Figure 1:**
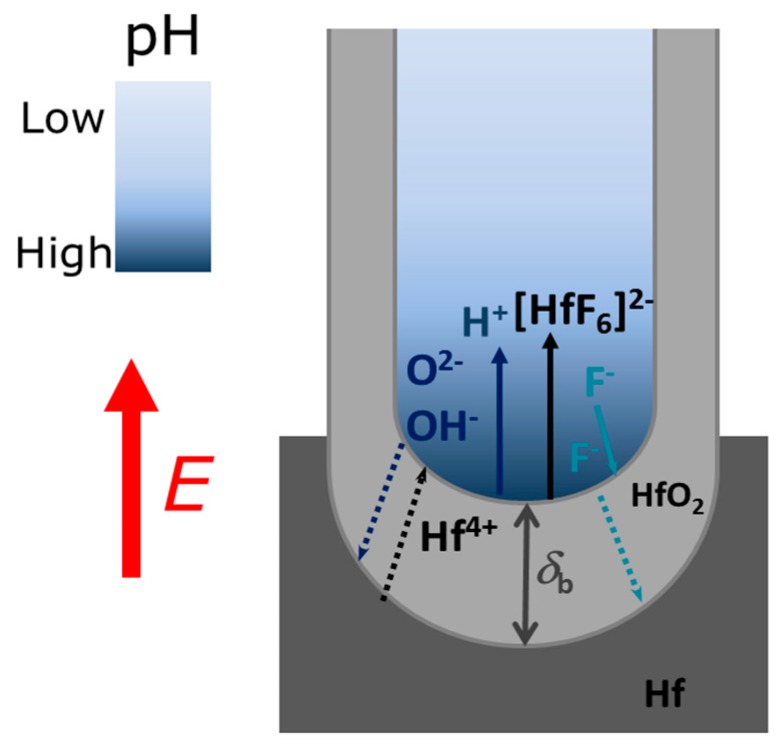
Scheme diagram illustrating the ions profiles inside the nanotubes (NTs) during the anodization.

The main mechanisms responsible for the formation of NTs by an Hf anodization processes are: (i) The electric field-assisted oxidation at the metal/oxide interface, forming an HfO_2_ continuous layer; (ii) the field-assisted dissolution of the oxide layer (at the oxide/electrolyte interface); and (iii) the chemical dissolution of the oxide by F^−^ ions at the metal/oxide and electrolyte/oxide interfaces (Figure 1). The electrochemical equations for HfO_2_ formation are:

Hf + 2H_2_O → HfO_2_ + 4H^+^ + 4e^−^ (1)

and

HfO_2_ + 6F^−^ + 4H^+^→ [HfF_6_]^2−^ + 2H_2_O (2)

for the oxidation (1) and dissolution (2) reactions, respectively (Figure 1). The reactions occurring at the anode are oxidation of the metal, that releases Hf^4+^ ions and electrons:

Hf → Hf^4+^ + 4e^−^ (3)

whereas, in the electrolyte one has the dissociation of water:

H_2_O → OH^−^ + H^+^ (4)

OH^−^ → O^2−^+ H^+^ (5)

Differently from the Al metal anodization case [25,26], where a steady-state condition is achieved (oxidation rate is balanced with the dissolution rate), the Hf anodization case (as that of Ti anodization) consists in a non-steady state anodization process with higher oxidation than dissolution rates [24]. Such effect severely compromises the HfO_2_ NTs length and growth [24]. There are also additional chemical dissolution effects during the anodization that affect the oxidation/dissolution equilibrium and limit NTs growth.

**Figure 2:**
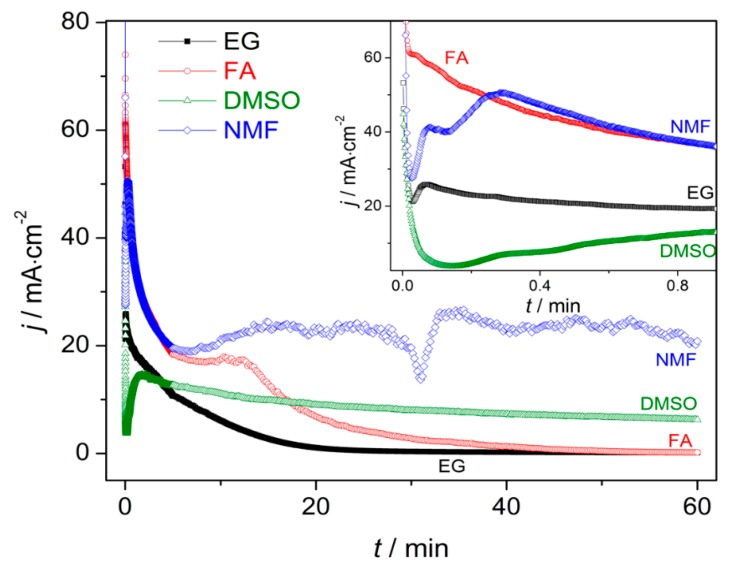
Current density anodization curves throughout the anodization [inset shows the transient period (1 min)] for ethylene glycol (EG), dimethyl sulfoxide (DMSO), formamide (FA) and N-methylformamide (NMF) samples.

**Figure 3:**
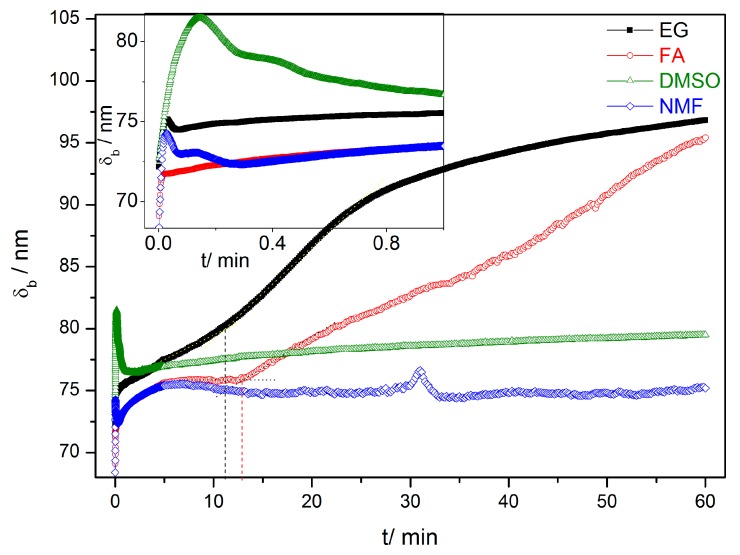
HfO_2_ barrier layer thickness (δ_b_) during the first hour of anodization for the EG, FA, NMF and DMSO samples.

The evaluation of the mechanisms that lead to the formation and growth of self-ordered HfO_2_ nanoporous/nanotubes, can be studied using current density [*j*(*t*)] curves (Figure 2) [23,24,25,26,27]. The evolution of the HfO_2_ barrier layer thickness (*δ*_b_) at the bottom of the NTs (Figure 1) was also estimated from the *j*(*t*) curves (Figure 3) [27]. According to the high-field conduction theory [28], the current density (*j*) is related to the voltage (*V*) drop across the barrier layer as follows:

(6) $$j\;=\;\alpha e^{\beta \frac{V}{\delta_{b}}}$$

where *α* and *β* are electrolyte and material-dependent constants and the (*V*/*δ*_b_) ratio is the effective electric-field across *δ*_b_ [27,28]. From Equation (6) we obtain,

(7) $$\delta_{b}\;=\;\frac{\beta V}{\ln\left(\frac{j}{\alpha }\right)}$$

during the anodization [24,27]. It was considered the material constants, *α* = 2.4 × 10^−9^ mA·cm^−2^ and *β* = 27.98 nm·V^−1^ (at room temperature), determined previously for TiO_2_ [27], and due to the physical similarities between these oxides here are also considered. Figure 3 shows the evolutions of *δ*_b_ along the anodization time calculated from the Equation (7).

*j*(*t*) curves for all the samples (EG, FA, NMF and DMSO; Figure 2 and corresponding inset) present the transient anodization characteristic of the successful formation of nanopore/nanotube arrays (as in the Al or Ti cases [23,24,25,26,27]). After applying 60 V, a continuous HfO_2_ layer is rapidly formed that leads to a resistance increase [rapid *j* decrease (inset of Figure 2) and *δ*_b_ increase (Figure 3)]. The following slight *j* decrease marks the initiation of pore nucleation, likely on the surface valley-type irregularities where the electric field enhances the oxide dissolution and promotes hole formation (i.e., the dissolution promoted by F^−^ ions in favorable spots of the HfO_2_ surface) [21,24]. Consequently, the HfO_2_ layer thickness starts to increase, while the pores/tubes formation accelerates. This is evidenced by the increase of *j* until a maximum is reached. A barrier layer, with thickness δ_b_, forms at the pores/tubes bottom (Figure 1 and Figure 3). Afterwards, the emerging porous structure will mechanically adjust and compete with each other in a self-organization process.

The differences between *j*(*t*) transient periods of each sample (inset of Figure 2) clearly reveals the decisive importance of the electrolyte solvent in promoting effective nucleation spots. In fact, comparing the *j*(*t*) transient period of each sample, we can observe three main aspects: (i) The lower *j*(*t*) values; (ii) the earlier emergence of NT nucleation; and (iii) the smaller nucleation time are attributed to samples FA, NMF, EG and DMSO, respectively.

As the anodization process evolves, *j*(*t*) of samples FA and EG present similar trends with the typical *j*(*t*)-decay of Ti anodization in fluoride-based electrolytes with EG [23,24,25,26,27]. This behavior arises from the non-equilibrium in the oxidation/dissolution processes, being the HfO_2_ dissolution lower than its formation, resulting in a slow decay of *j*(*t*) during the anodization. As a result, a progressive increase of δ_b_ of the NTs’ occurs, as shown in Figure 3. Consequently, the ionic migration path along the oxide barrier [27,29] significantly extends, inhibiting the transport of F^−^, Hf^4+^ and O^2−^ ions across δ_b_ (Hf^4+^ and O^2−^ for oxidation, F^−^ for dissolution) which subsequently limits a further NTs growth (Figure 1). Additionally, chemical effects, such as local pH decrease, occur throughout the anodization leading to the chemical dissolution of the NTs wall preferentially at the NTs tops (V-shape NTs) [17,18,27,29,30]. The NMF *j*(*t*) curve presents a large decay up to 8 min, similar to EG and FA, but then an overall constant *j*(*t*) emerges, although with some singularities during the anodization.

On the other hand, in the case of the DMSO, the *j*(*t*) curve remains approximately constant throughout the anodization, indicating a more optimized anodization for NTS growth, with balanced oxidation/dissolution processes that lead to a constant oxide growth rate (similar with Al nanoporous anodization were no limit in length is imposed [25,26]). Additionally, the DMSO *j*(*t*) transient curve shows an extended nucleation period of time (indication of low-rate pore nucleation) [24] with fairly smaller *j*(*t*) values over such region [indicating a thinner δ_b_ (Figure 3)] when comparing with other samples (FA or EG) [24,27].

Additionally, Figure 3 shows that by changing the electrolyte medium, we obtain different final δ_b_. Furthermore, for each sample, the capacitance density (C) at the oxide barrier was also calculated from the *j*(*t*) curves [Supporting Information (SI) - Figure S3 and Table S1]. At the end of the anodization (1 h), we can extract the final δ_b_ and C for each sample (SI - Table S1). It shows that the FA/EG samples led to thicker δ_b_ (and lower C) and DMSO/NMF led to thinner δ_b_ (and higher C).

Moreover, we perform additional anodizations with the same conditions as for the samples EG, FA, NMF and DMSO during 17 h (SI - Figure S1). In this case, *j*(*t*) of the sample NMF rapidly decays after 444 min. This corresponds that a complete conversion on the Hf foil into HfO_2_ has occurred at this time (444 min), as observed by SEM cross-section images (SI - Figure S2).

### 3.2. Growth Rate with Different Organic Solvents

**Figure 4:**
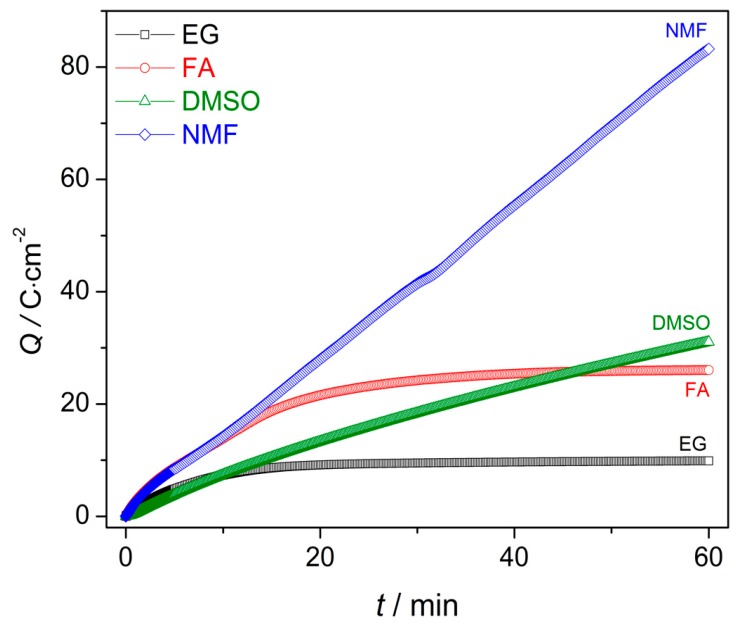
Charge curves during the first hour of anodization for the EG, FA, NMF and DMSO samples.

Figure 4 shows the charge curves *Q*(*t*) obtained from the integration of the *j*(*t*) data. The *Q*(*t*) curves describe the growth rate along the anodization [24]. Until the first 13 min both *Q*(*t*) slopes of NMF and FA samples are higher than those of the DMSO and EG. Although the NMF sample presents a higher charge over time for the entire anodization period (comparing to the rest of the samples), *Q*(*t*) of the DMSO sample overcomes that of the FA sample at the end of the anodization period (close to 50 min). Higher *Q*(*t*) curve indicates higher charge transfer, leading to a higher growth rate. The charge transferred during the anodization process can then be related to the solvent characteristics and *Q*(*t*) values. Additionally, the *Q*(*t*) curves present different slope’s trend: Whereas, in NMF and DMSO samples the slope is fairly linear, providing an almost constant AHO growth rate, that is not the case for the EG and FA samples, where *Q*(*t*) has a non-linear slope and presents two distinct growth rate regimes. After 11 min and 13 min of anodization time, for EG and FA, respectively, the growth rate slows down. With these electrolytes, the δ_b_ increases during the anodization, leading to a constant *Q*(*t*) over time [and significantly lower final *Q*(*t*) values]. As discussed before, the δ_b_ increase is related to the unbalanced oxidation-dissolution rate reactions, being the HfO_2_ dissolution smaller than its formation, ultimately limiting the NTs growth and length [24,27]. δ_b_(*t*) curves (Figure 3) of NMF/DMSO samples present thinner δ_b_, while EG/FA samples shows thicker δ_b_. One can observe the similar *Q*(t) trend of the two groups of samples EG/FA and NMF/DMSO. EG and FA presents the transition of two regimes at 11 min and 13 min, respectively. After these anodization times, δ_b_ greatly increases (Figure 3) and the growth rate slows down. On the other hand, NMF/DMSO samples show a *Q*(*t*) linear slope, corresponding to a constant δ_b_ over time (Figure 3). At the end, EG/FA samples presents thicker δ_b_ than NMF/DMSM samples.

### 3.3. Morphology and Layer Thickness 

**Figure 5:**
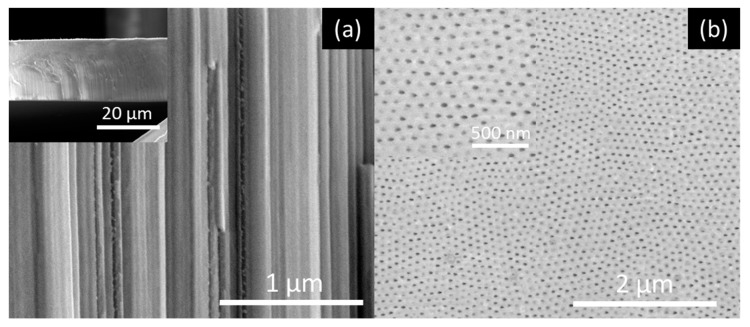
SEM images of the nanoporous AHO templates after 1 h of anodization for the FA sample: (**a**) Cross-section view (inset at lower magnification) and (**b**) top-view (inset at higher magnification).

**Figure 6:**
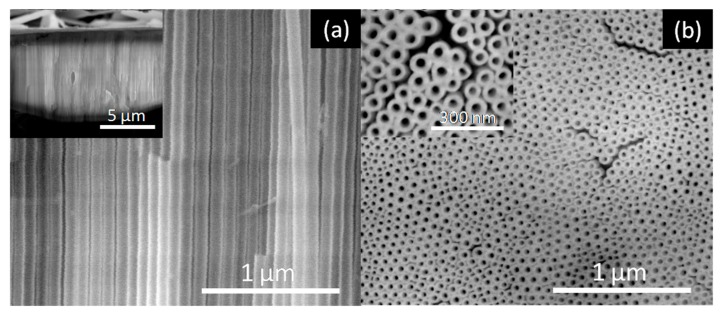
SEM images of the AHO NTs after 1 h of anodization for the EG sample: (**a**) Cross-section view (inset at lower magnification) and (**b**) top-view (inset at higher magnification).

**Figure 7:**
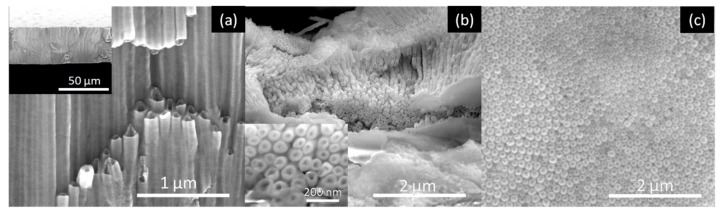
SEM images of the AHO NTs after 1 h of anodization for the DMSO sample: (**a**) Cross-section view (inset at lower magnification), (**b**) top view (inset at higher magnification) and (**c**) bottom view.

**Figure 8:**
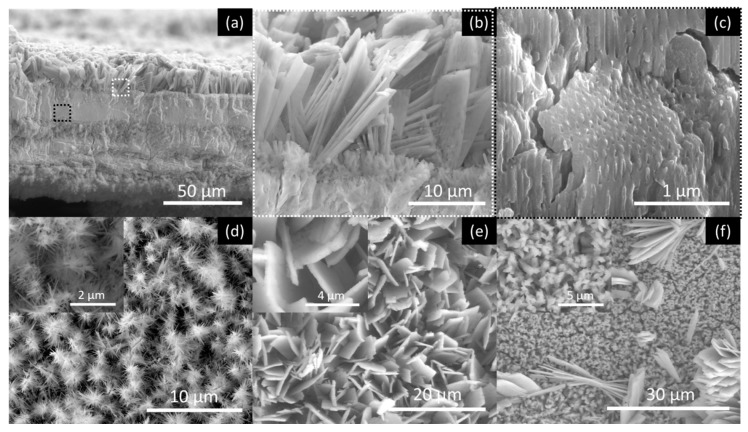
SEM images of the AHO nanostructures after 1 h of anodization for the NMF sample. Cross-section images showing (**a**) the thick oxide layer that contains (**b**) nanoflakes at the layers’ top and (**c**) self-ordered nanoporous structures (zone areas where (**b**,**c**)) images where extracted from (**a**) images are indicated; top view shows (**d**) nanoneedles (inset at higher magnification) (**e**) nanoflakes (inset at higher magnification) and (**f**) nanowires-agglomerations (inset at higher magnification), all present in this sample.

Figure 5, Figure 6, Figure 7 and Figure 8 shows SEM cross-section, and top view images of the AHO templates for all samples after 1 h of anodization. Comparing the different samples, one sees that the electrolyte solvent has a critical impact on the AHO morphology, growth rate and layer thickness. From the top view images, we can see that FA leads to a self-ordered nanoporous template (Figure 5), while EG and DMSO lead to highly self-ordered NT arrays with hexagonal closely packed distribution (Figure 6 and Figure 7, respectively). For the NMF samples (Figure 8), instead of homogeneous NTs or nanoporous structures, we obtained different morphologies, ranging from Figure 8a a continuous oxide layer, Figure 8b nanoporous, Figure 8c nanoneedles, Figure 8d nanoflakes or Figure 8e agglomerated nanowires.

EDS analyses showed (SI-Figure S5) that the anodic as-grown nanoporous (FA) nanotubes (DMSO/EG) presents significant amounts of F, which is typical of anodic HfO_2_ or TiO_2_ structures [14,18]. Literature associates the F presence with the formation of hafnium oxyfluoride in the AHO. For the sample NMF the same the F presence was obtained (SI - Figure S5d). Additionally, in NMF sample we perform separated EDS analyses for bulk oxide (SI - Figure S5e: Z1 area), and surface top nanostructures (flakes/needles; SI - Figure S5f: Z2 area) identifying the same chemical elements in both areas and are in accordance with the other anodic hafnium oxide samples.

**Figure 9:**
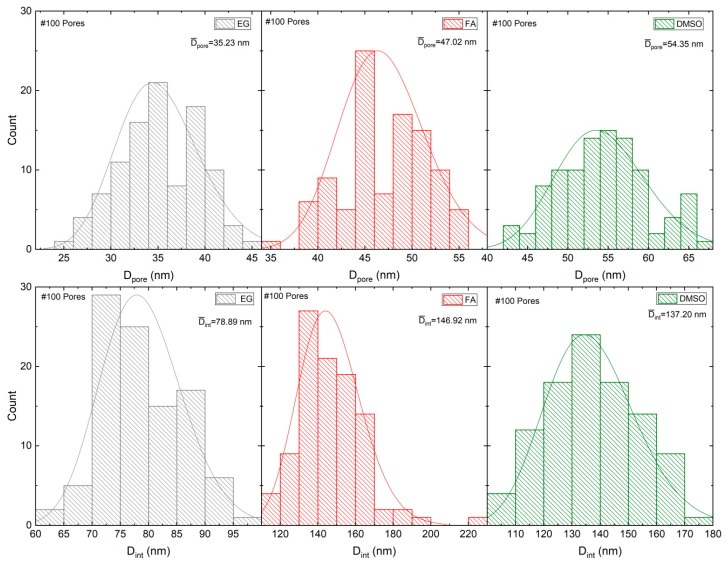
Histograms of the size distribution, *D*_p_ and *D*_int_, which were fitted with a normal distribution for samples EG, FA and DMSO.

The pore diameter (*D*_p_), and interpore distance (*D*_int_) geometrical parameters were extracted from the SEM image (100 pores analyzed) for the EG, FA and DMSO samples as shown in Table 1. The average *D*_p_ and *D*_int_ (and standard deviation SD) were estimated from the histogram of the size distribution, which were then fitted to a normal distribution (Figure 9).

Usually, the regularity of the geometrical patterns in self-ordered nanoporous/nanotubes of anodic TiO_2_, Al_2_O_3_ or HfO_2_ is analyzed by a typical parameter—the porosity (*P*). For the well-defined hexagonal porous structures, *P* of the anodic oxide layer is given by the equation proposed by Nielsch et al.:

(8) $$P\;=\;\frac{2\pi }{\sqrt{3}}{\left(\frac{r}{D_{int}}\right)}^{2},$$

where *r* is the pore radius (*r* = D_p_/2) [31]. For the hexagonal self-ordered nanoporous Al_2_O_3_, or TiO_2_ NT arrays the obtained porosities are close to 10% (10% porosity rule) when mild anodizations are implemented (low anodization potentials). This rule assumes that a perfect hexagonal structure shows a *P* of 10% and deviations from these values results in the imperfect ordering of the structures. Qiu et al. presented a study of porosity for anodic HfO_2_ NTs (electrolyte with ethylene glycol as solvent) and obtained porosity values of 10% when the anodizations were performed within the range of 10 to 40 V [14]. In this work, *P* was calculated for the samples FA, EG and DMSO, as shown in Table 1. For the FA and DMSO samples, porosities closer to 10% (of ~9.3 and 14.2%, respectively) were obtained, consistent with the 10% rule. However, for the EG sample *P* clearly deviates from the 10% rule (*P*~18.1%).

The resulting AHO layer thicknesses are shown in Table 2. The EG, FA and DMSO samples have a mean AHO layer thickness (*L*) of approximately 8.0; 23.6 and 37.3 µm, respectively. On the other hand, the NFM sample shows a rapid AHO growth rate with *L* = 94.8 µm, much larger than the other samples. Notice that while previously discussing the *Q*(*t*) curves (Figure 3), the higher final *Q*(*t*) was indicative of thicker *L*: *L*(NMF) > *L*(DMSO) > *L*(FA) > *L*(EG), as obtained [24].

### 3.4. Electrolyte Solvent as the Driven Factor behind AHO Morphology, Porosity and Growth

In this study, there are two relevant parameters in the electrolyte solvent: The viscosity (η) and dielectric constant (κ) (Table 2). The *Stokes-Einstein* equation relates the diffusion constant (*D*) of a macroscopic particle of radius *r*, undergoing a Brownian motion, to the viscosity η of the fluid in which it is immersed [32]. Thus, at a constant temperature, the individual ions [O^2−^] or [F^−^] diffusion constant will be inversely dependent on solvent η, limiting both oxidation and dissolution rates. On the other hand, a high-κ solvent draws a higher electrolyte capacitance (for a constant potential) inducing the formation of more charges at the oxide layer, thus, improving the extraction of the Hf^4+^ ions and ultimately leading to a high oxidation rate [17,18]. Therefore, κ will be intimately related to the rate of the oxidation processes [Equation (1)] at the oxide/metal interface (higher κ, higher oxidation rate).

**Figure 10:**
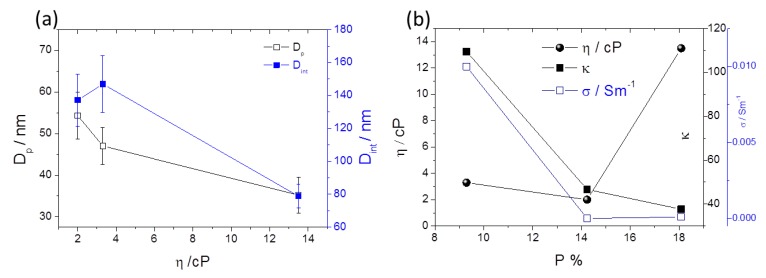
(**a**) *D*_p_, *D*_int_ as a function of the solvent viscosity η and (**b**) η and κ;σ as a function of the porosity (*P*).

Figure 10a,b shows the analyses of the features *D*_p_, *D*_int_ and *P* as a function of the solvent physical parameters, η and κ. *D*_p_ and *D*_int_ decrease as η increases (Figure 10a). The individual ions [O^2−^] or [F^−^] diffusion constant will be inversely dependent on solvent η (according to Stokes-Einstein equation), limiting both oxidation and dissolution processes rates. Increasing η, the diffusion of [O^2−^] or [F^−^] will decrease, leading to smaller *D*_p_ (and *D*_int_) (Figure 10a) and higher *P* (Figure 10b) (deviating from the optimized 10% rule for optimized self-ordered regularity). On the other hand, by decreasing κ [or the solvent conductivity (σ) SI: Table S1], *P* increases, also leading to deviations of 10% rule). 

**Figure 11:**
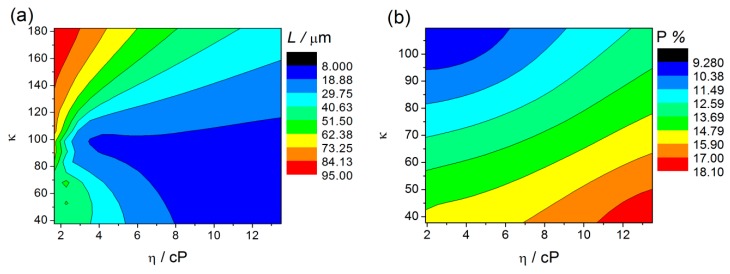
Counterplots of *L* (**a**) and *P* (**b**) as a function of the solvent parameters η and κ, obtained by interpolation (cubic Spline-20 points) of η, κ, *L* and *P* values.

Figure 11a,b displays the counterplots (color-maps) of the AHO *L* and *P* as a function of the solvent physical parameters η and κ. We used the parameters for each sample, (η, κ, *L*) from Table 2 and (η, κ, *P*) from Table 1 and Table 2, to perform a numerical estimation of 20 new data points by the interpolation method of cubic Spline. By this interpolating method, we are able to create an estimation of new values of *L* and *P* with certain conditions of the electrolyte (varying η and κ). The obtained (η, κ, *L*) and (η, κ, *P*) arrays were then plotted in 3D counterplot in Figure 11a,b, respectively. One can observe that thicker AHO is obtained for high κ and low η values. These anodization conditions led to extremely fast oxidation rates as in the case of the NFM sample and demonstrated by the *Q*(*t*) curve (Figure 4). The NFM sample shows a faulty structure without self-ordered nanoporous or tubes [only small areas revealed a nanoporous structure; Figure 7c]. Indeed, much faster oxidation than field-enhanced dissolution occurred during the anodization, being the process out of the steady-state anodization conditions, which is mandatory for nanoporous/tubes upraise. We believe that, at the initial anodization stages, already nanoporous/NTs formation occurred [see initial *j*(*t*) transient in Figure 2 and SEM image in Figure 8c], but the NMF solvent high-κ (Table 2) led to a much faster Hf^4+^ extraction, and thus, leaving no time to maintain the nanostructures self-organization regime, i.e., to have a proper dissolution rate that would counter-balance the high oxidation rate. Additionally, the NMF *j*(*t*) singularities observed during the anodization (Figure 2) can be related to the different nanostructures morphologies obtained (Figure 8). 

Figure 11b shows the counterplot of *P* as a function of the solvent parameters η and κ. With this analysis, we establish a range of *P* tunability. We can observe that porosities closer to the 10% rule can be obtained for higher κ and lower η. However, it is also observed that the porosity clearly deviates from the 10% rule for lower values of κ and higher η. Notice that moderate values of *P* (closer to 10%) can be obtained with higher η values, but κ has to be at the higher value range. Contrarily to what is usually presented in literature, that *P* depends exclusively on anodization parameters such, voltage, (*D*_p_, *D*_int_) or water content, we demonstrate for the first time that the porosity also critically depends on physical properties of the solvent (η, κ). These new results bring the possibility to mix the solvents in order to tune the anodic oxides with a perfect hexagonal arrangement. 

The anodization conditions mandatory for the self-ordered nanoporous/tubes morphology to arise are obtained decreasing κ, either with low or high η. The FA solvent has a relatively lower κ, but a slightly higher η than those of NMF. This seems to be enough to establish the necessary conditions of a more equilibrated oxidation/dissolution balance for the formation of structures with self-organization (nanoporous structure; Figure 5). Additionally, under these conditions, moderate *L* is obtained, as shown in the counterplot map (Figure 11a). On the other hand, the NT structure arises when decreasing even more κ, either in low or high η regimes, although with thinner or thicker thicknesses, respectively. Both DMSO and EG samples showed an NT structure, but the DMSO sample presented a higher *L*. Both own a similar κ value, albeit much smaller than the one from the previously discussed samples. EG much higher viscosity, making F^−^ ions more difficult to be replaced by new ones at the NTs bottom. As a result, oxidation is faster than dissolution, leading to a *j*(*t*) decrease during the anodization, indicating the progressive increase of δ_b_. Thus, the ionic migration path along the oxide barrier [29] significantly extends, inhibiting the transport of F^−^, Hf^4+^ and O^2−^ ions across δ_b_ (Hf^4+^ and O^2−^ for oxidation, F^−^ for dissolution) which subsequently limits a further NT growth (Figure 1). On the other hand, from the dissolution reaction [Equation (2)] one can see that the failure of F^−^ leads to H^+^ excess, and thus, to additional chemical dissolution effects that also result in limited NTs growth. DMSO showed a perfect balance between oxidation and dissolution, *j*(*t*) constant during the anodization, just as in the Al anodization case [23,25,26]. The increased NT-array length when using a DMSO electrolyte can also be attributed to the controlled chemical dissolution process effect. Thus, the route to successfully obtain long NT arrays is to minimize the pH decrease promoted by H^+^ additional etching. The DMSO aprotic photophilic solvent accepts an H^+^ ion from NH_4_F and reduces its activity, decreasing the chemical etching. Thus, allowing the DMSO NTs to grow deep into the hafnium foil without any significant loss at the tube tops. The presence of DMSO modifies the space charge region in the pores, thereby also avoiding the lateral etching and leading to a steady-state pore growth and low chemical etching of the NT walls.

As expected, the same conclusions were obtained for the counterplots with the solvent conductivity (σ) instead of using κ (SI - Figure S6).

**Figure 12:**
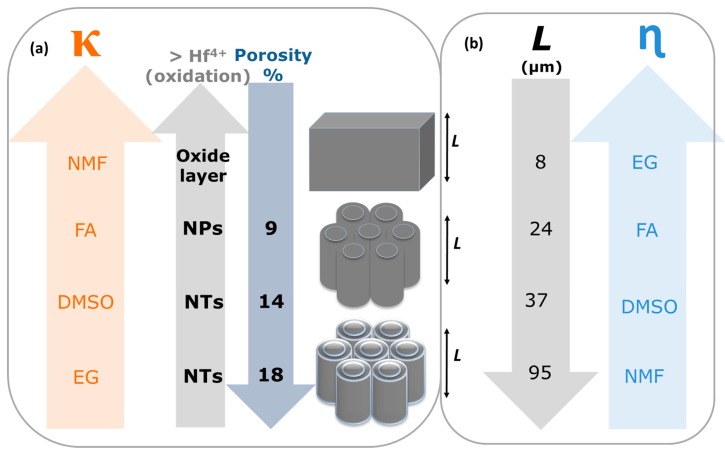
Scheme of (**a)** AHO morphology transition from bulk-to-nanoporous (NPs)-to-nanotubes (NTs) with the electrolyte solvent constant dielectric constant (κ) increase (as the porosity (*P*) decreases); and (**b**) the AHO layer thickness (*L*) increase with the viscosity (η) decrease.

In summary, the electrolyte solvent affects the morphology and length of anodic HfO_2_ (Figure 12). Different nanostructures with different shapes of morphologies are obtained by changing the electrolyte solvent physical characteristics. For instance, we can tailor the anodic oxide morphology from NTs (EG and DMSO) to nanoporous (NPs; FA) to a thick oxide layer (NMF), by increasing the electrolyte κ, since the oxidation rate is higher (Figure 11a (easier Hf^4+^ extraction). Additionally, with the κ increase the porosity decreases (Figure 11a). On the other hand, increasing η, the *L* severally decreases (Figure 11b), since the dissolution rate decreases because the ionic diffusion is limited. Overall, an accurate balance between the electrolyte solvents’ κ and η is needed to obtain the desired morphology, porosity and length.

## 4. Conclusions

We investigated the growth of self-ordered anodic hafnium oxide (AHO) by using different solvent base electrolytes: EG, FA, NMF and DMSO. We found that the solvents are a key factor for tunning the possible morphology of the nanostructures of the AHO. EG and DMSO allow vertically oriented growth in self-ordered NT arrays, due to low κ and high η (in the case of EG), and because the photophilic character (in the case of DMSO) of the solvent. On the other hand, FA and NMF lead to nanoporous AHO (for FA), due to their much higher κ, and to diverse nano-shapes (for NMF), including nanoflakes, nanoneedles, nanotube-agglomerations and thick continuous oxide. Furthermore, the final layer thickness of the AHO was also correlated with the electrolyte solvent type, and particular its κ and η values. While κ determines a higher oxidation rate (out of the steady state regime), leading to thicker HfO_2_ oxide layer (NMF) out of the self-organization anodization regime (nanoporous or nanotubes), a lower κ combined with lower η lead to higher lengths, but in the self-ordered regime, and thus, to hexagonally distributed NTs (DMSO). Additionally, porosities within the 10% self-ordered regime were obtained for high κ and low η.

The detailed analyses of *j*(*t*), δ_b_(*t*) and *Q*(*t*) anodization curves combined with morphology analyses demonstrate that an accurate balance between the oxidation and dissolution rates during the anodization is mandatory to obtain optimized self-ordered nanostructures. The anodization curves *j*(*t*) and *Q*(*t*) for the different solvent electrolytes revealed different growth mechanism and growth rates of AHO. 

Overall, the κ and η constants from the solvent electrolyte directly affects the transition from thick oxide-to-nanoporous-to-nanotubes (as κ decreases), the porosities and the growth of oxide layer thickness, (as η increase). This study clearly reveals that the organic solvent is the main factor affecting the transition from pores to tubes and the regularity of the structures, as well as the anodization growth rates.

## Figures and Tables

**Table 1:** AHO nanoporous and nanotubes geometrical parameters: pore diameter (*D*_p_) interpore distance (*D*_int_) and Porosity (*P*) for the different samples.

Sample	*D*_p_(nm)	*D*_int_ (nm)	*P%*
FA	47.02	146.92	9.3
EG	35.23	78.89	18.1
DMSO	54.35	137.2	14.2

**Table 2:** Solvent characteristics: Classification at room temperature, dielectric constant (κ) and viscosity (η); AHO mean layer thickness *L* and the type of nanostructures obtained for the different samples.

Sample	Classification	κ	η (cP)	*L* (µm)	Nanostructure
EG	Polar protic	37.70	13.50	8	Nanotubes
FA	Polar protic	109.5	3.302	23.7	Nanoporous
DMSO	Polar aprotic	46.70	1.996	37.3	Nanotubes
NMF	Polar protic	182.4	1.650	94.8	Several

## Supplementary Materials

The following are available online at https://www.mdpi.com/2079-4991/10/2/382/s1, Figure S1: Current density anodization curves during 17 h, Figure S2: SEM images of the NMF sample for 17 h of anodization, Figure S3: Capacitance Calculation Estimative - Capacitance density as a function of the anodization time (60min), Figure S4: Barrier layer thickness (*δ*_b_) and capacitance density (*C*), at the AHO nanotubes/nanoporous bottom, as a function of the electrolyte viscosity (η), Table S1: Summary of electrolyte physical parameters (κ, η and σ) and the AHO experimental parameters extracted from the anodization curves (δ_b_ and C) and from the SEM images (*L* and *P*), Figure S5: EDS Spectroscopy—Chemical Characterization - EDS spectra for all the samples, Figure S6: Counterplots of L and P as a function of the solvent parameters η and conductivity (σ).
