# Supplementary material for: Tailoring the Anodic Hafnium Oxide Morphology Using Different Organic Solvent Electrolytes
Source: Nanomaterials (Basel). 2020 Feb 22;10(2):382. doi: 10.3390/nano10020382 (PMC7075290; doi:10.3390/nano10020382)
Supplement: Supplementary file 1 [file nanomaterials-10-00382-s001.pdf]

Supplementary Materials: Tailoring the anodic hafnium oxide morphology using different organic solvent electrolytes

Arlete Apolinário [1,*], Célia T. Sousa [1], Gonçalo N. P. Oliveira [1], Armandina M. L. Lopes [1], João Ventura [1], Luísa Andrade [2], Adélio Mendes [2] and João P. Araújo [1,*]

[1] Instituto de Física de Materiais Avançados, Nanotecnologia e Fotónica (IFIMUP), Departamento de Física e Astronomia, Faculdade de Ciências, Universidade do Porto, Rua do Campo Alegre, 678, 4169-007 Porto, Portugal; celiasousa@fc.up.pt (C.T.S.); goliveira@fc.up.pt (G.N.P.O.); armandina.lopes@fc.up.pt (A.M.L.L.); joventur@fc.up.pt (J.V.)

[2] Laboratory for Process Engineering, Environment, Biotechnology and Energy (LEPABE), Departamento Engenharia Química, Faculdade de Engenharia, Universidade do Porto, R. Dr. Roberto Frias, 4200-465 Porto, Portugal; luisa.andrade@fe.up.pt (L.A.); mendes@fe.up.pt (A.M.)

* Correspondence: arlete.apolinario@fc.up.pt or arletteapolinario@gmail.com (A.A.); jearaujo@fc.up.pt (J.P.A.); Tel.: +351-220402334 (J.P.A.)

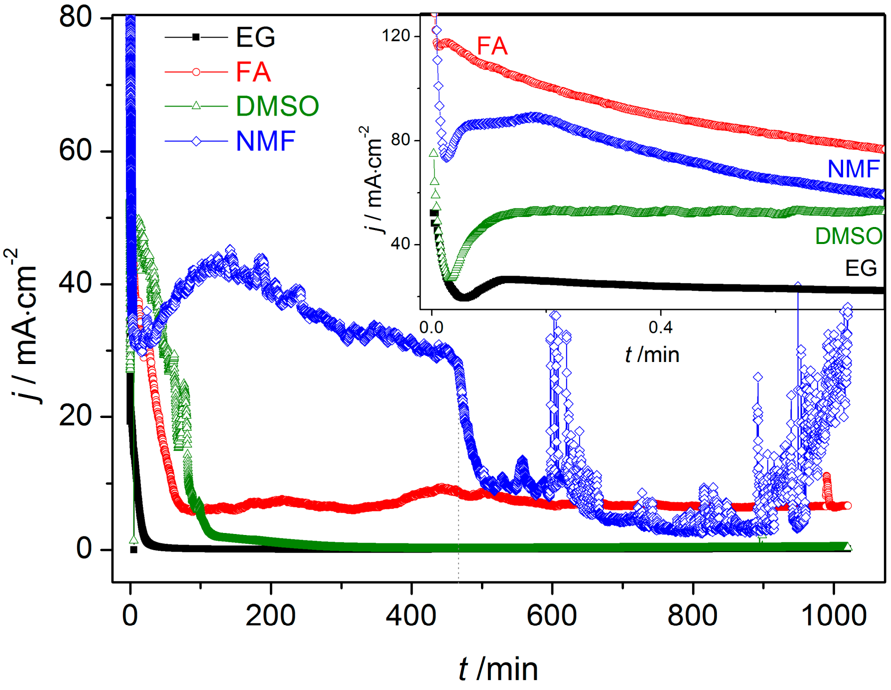

Figure S1. Current density anodization curves during an anodization of 17 h (inset shows the transient period) for all samples. We can observe that the $j(t)$ of the sample NMF rapidly decays at the anodization time 444 min. This corresponds to that the complete conversion on the Hf foil into HfO_2 occurred at this time.

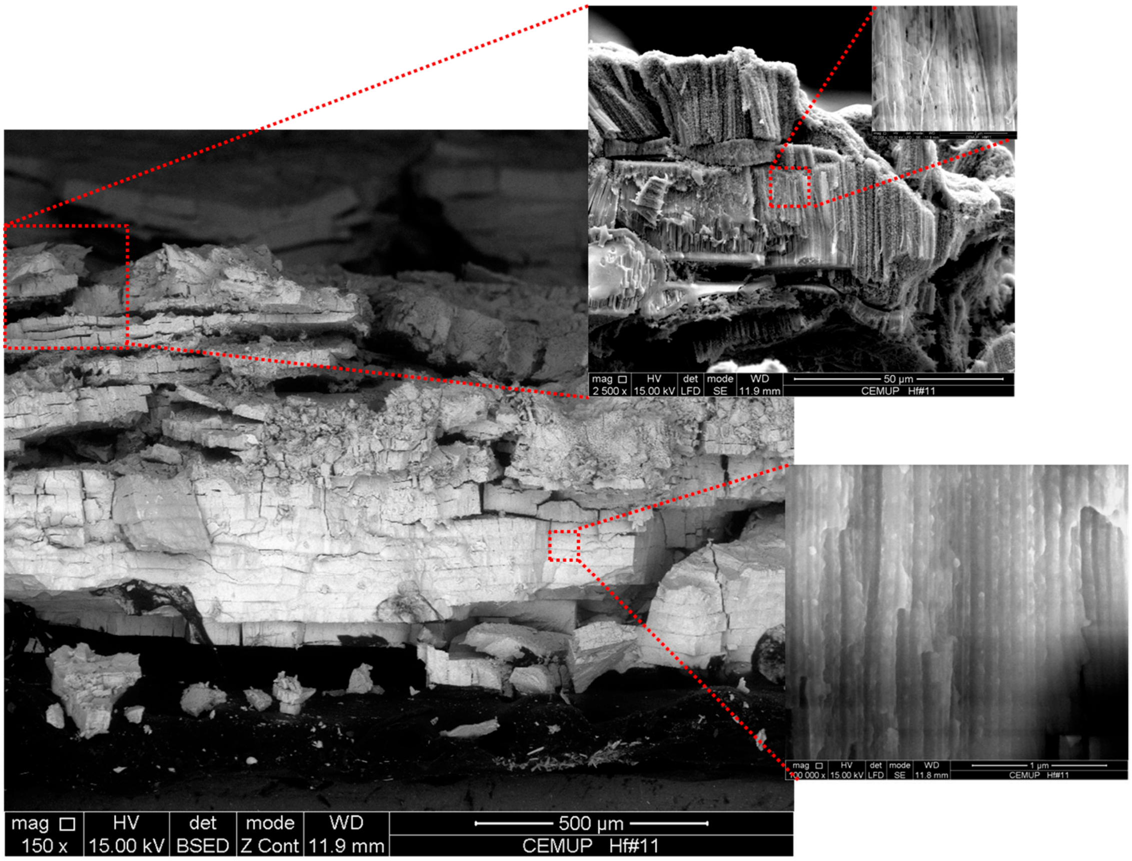

Figure S2. SEM images of the AHO nanostructures after 17 h of anodization for the NMF sample. Cross-section images shows a thick oxide layer and self-ordered nanoporous structures. With the NMF electrolyte a complete conversion of the Hf foil was converted into HfO_2. The anodization curves shows that the oxides is completely converted at approximately 444 min (the rapid noisy decay of the current density is indicative of the electrolyte leak since all the foil was anodized

Capacitance Calculation Estimative.

The capacitance, C was obtained after calculating the barrier layer thickness considering the high-field conduction theory. By considering, for approximation, the bottom of the nanotube a parallel plate condenser with a dielectric constant κ′(dielectric constant of the oxide) between the plates :

$$E = \frac{V}{\delta_b} = \frac{Q}{\varepsilon_0 \kappa' A} \qquad \text{Equation (S1)}$$

$$C = \frac{Q}{V} = \frac{\varepsilon_0 \kappa' A}{\delta_b} \qquad \text{Equation (S2)}$$

(where *E*, is the electric field; *V*, the anodization voltage; *Q*, the charge; *C*, the capacitance; *A*, the area; δ_b, the barrier layer thickness; $\varepsilon_0 = 8.85 \times 10^{-12}$ ($F \cdot m^{-1}$) is the vacuum permittivity and κ′ the dielectric constant of the material (HfO_2). For HfO_2, κ′ = 25, the anodization voltage was *V* = 60 V and considering *A* approximately the anodization area, we can obtain an estimative of the capacitance density (F/cm^2) at the bottom of the tubes/pores. Note that even if the effective area changes during the anodization, its impact on the extracted value δ_b is small.

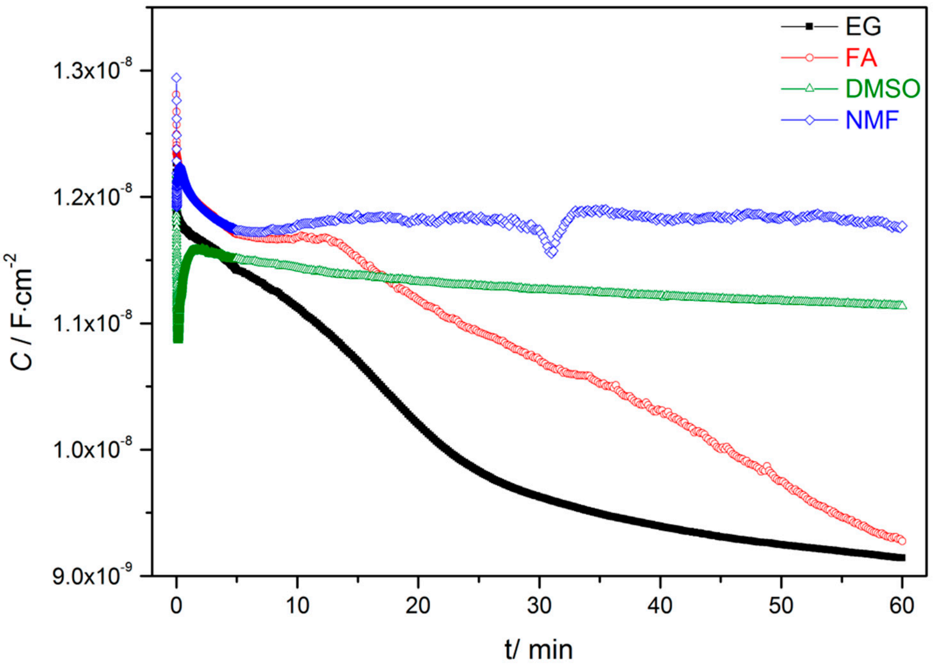

Figure S3. Capacitance density during the anodization of 60 min for all samples.

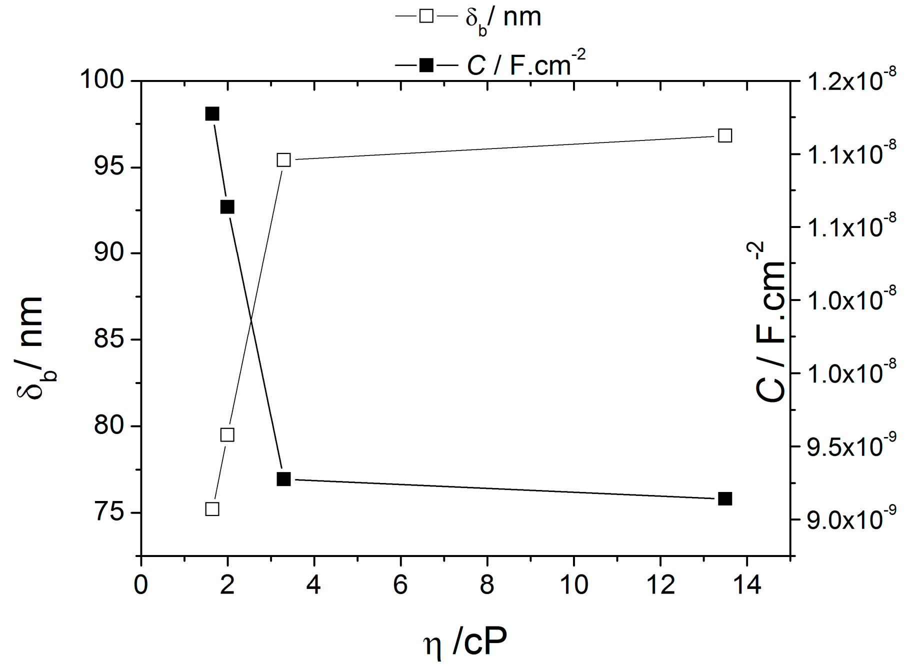

Figure S4. Barrier layer thickness (δ_b) and capacitance density (C), at the AHO nanotubes/nanoporous bottom, as a function of the electrolyte viscosity (η) for all samples. The δ_b and C values were extracted from the anodization of 60 min. We can see that by increasing the electrolyte η, δ_b increases [as C decreases according to Equation (S2)].

Table S1. Summary of electrolyte physical parameters (κ, η and σ) and the resulting anodic HfO_2 experimental parameters extracted from the anodization curves (δ_b and *C*) and from the SEM images (*L* and *P*).

Sample	κ	η (cP)	σ ($S\cdot m^{-1}$)	δ_b (nm)	C ($F\cdot cm^{-2}$)	*L* (μm)	P %
EG	37.70	13.50	1.0×10^{-4}	96.83	9.14×10^{-9}	8.00	9.3
FA	109.5	3.302	1.0×10^{-2}	95.42	9.28×10^{-9}	23.7	18.1
DMSO	46.70	1.996	3.0×10^{-6}	79.50	1.11×10^{-8}	37.3	14.2
NMF	182.4	1.650	3.0×10^{-2}	75.20	1.18×10^{-8}	94.8	9.3

EDS Spectroscopy—Chemical Characterization.

Figure S5 shows the EDS spectra for all the samples. For all the samples EDS analysis shows that the anodic as-grown nanoporous (FA) nanotubes (DMSO/EG) presents significant amounts of F typical of the anodic HfO_2 NTs or TiO_2 NTs [1, 2]. As referred in the literature, this indicates the formation of hafnium oxyfluoride in the anodic hafnium oxide. Typically, the fluorine content decreases or is completely removed after a high temperature thermal annealing [1].

For the sample NMF we perform separated EDS analyses for bulk oxide (Figure S5e Z1 area) and surface top nanostructures (flakes/needles; Figure S5f Z2 area) being identify the same chemical elements in both areas and are in accordance with the other anodic hafnium oxide samples.

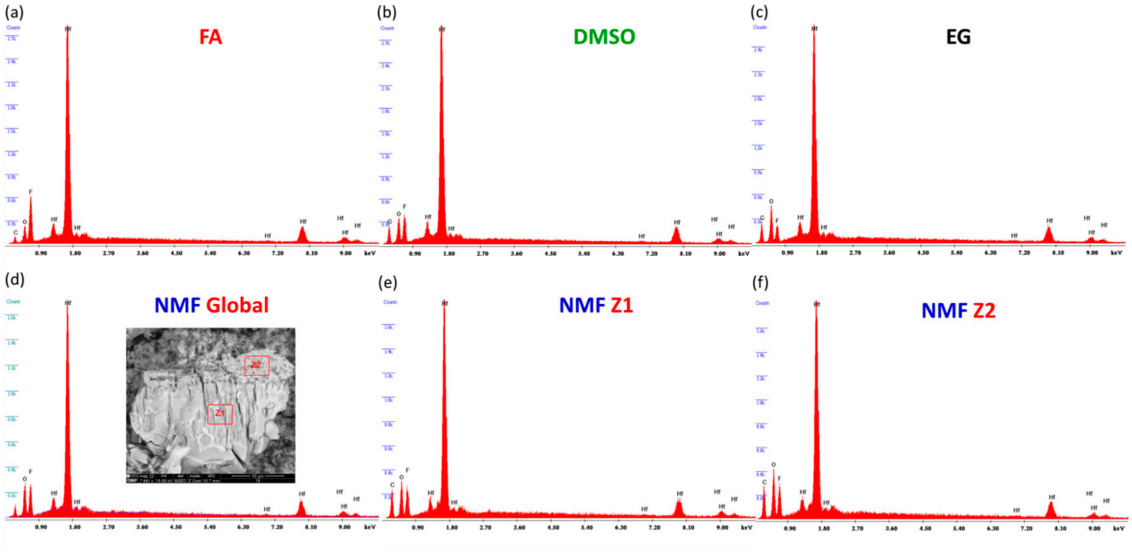

Figure S5. EDS spectra for the samples FA (a), DMSO (b), EG (c) and NMF (d-f). NMF sample was analyzed in a global area at the top (d) and in separated areas: at the top (e; Z2: nanoflakes/nanoneedles) and at the bulk thick oxide (f; Z1) as indicated in SEM image of (d).

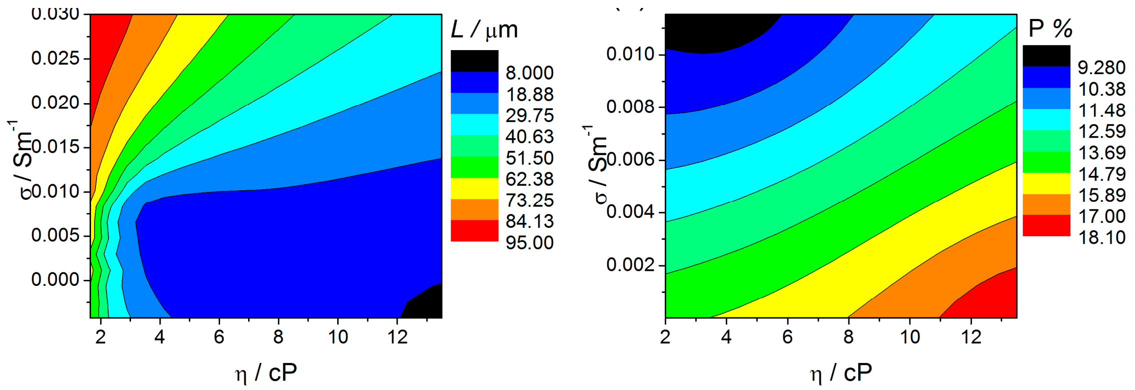

Figure S6. Counterplots of *L* (left) and *P* (right) as a function of the solvent parameters η and conductivity (σ), obtained by interpolation of η, σ, *L* and *P* values.

References

1. Qiu, X.; Howe, J. Y.; Cardoso, M. B.; Polat, O.; Heller, W. T.; Parans Paranthaman, M., Size control of highly ordered HfO2nanotube arrays and a possible growth mechanism. *Nanotechnology* **2009,** 20, (45), 455601.
2. Shankar, K.; Mor, G. K.; Prakasam, H. E.; Yoriya, S.; Paulose, M.; Varghese, O. K.; Grimes, C. A., Highly-ordered TiO2nanotube arrays up to 220 μm in length: use in water photoelectrolysis and dye-sensitized solar cells. *Nanotechnology* **2007,** 18, (6), 065707.
